# A low-carbohydrate, high-fat diet leads to unfavorable changes in blood lipid profiles compared to carbohydrate-rich diets with different glycemic indices in recreationally active men

**DOI:** 10.3389/fnut.2024.1473747

**Published:** 2024-10-16

**Authors:** Anna Maria Kripp, Andreas Feichter, Daniel König

**Affiliations:** ^1^Department of Nutritional Sciences, Faculty of Life Sciences, University of Vienna, Vienna, Austria; ^2^Vienna Doctoral School of Pharmaceutical, Nutritional, and Sport Sciences, University of Vienna, Vienna, Austria; ^3^Department of Sport Science, Centre for Sport Science and University Sports, University of Vienna, Vienna, Austria

**Keywords:** blood lipids, cholesterol, lipoproteins, triglycerides, carbohydrates, low-carb, glycemic index

## Abstract

**Objective:**

In addition to recent discussions of low-carbohydrate, high-fat diets (LCHF) from a performance perspective, there is a paucity of knowledge regarding influence of the combined effect of an exercise and nutritional intervention, which varies in carbohydrate (CHO) intake and glycemic indices, on blood lipid levels in recreationally active men.

**Methods:**

A total of 65 male runners (VO_2_ peak = 55 ± 8 mL·min^−1^·kg^−1^) completed a 10-week *ad libitum* nutritional regimen (LOW-GI: ≥ 65% low GI CHO per day, *n* = 24; HIGH-GI: ≥ 65% high GI CHO per day, *n* = 20; LCHF: ≤ 50 g CHO daily, *n* = 21) with a concurrent prescribed endurance training intervention. Fasting total cholesterol (TC), triglycerides (TG), low-density lipoprotein cholesterol (LDL-C) and high-density lipoprotein cholesterol (HDL-C) were determined before and after the intervention. Additionally, 24-h dietary recalls were completed twice weekly.

**Results:**

Following the intervention, TC was significantly higher in LCHF (196 ± 37 mg·dL^−1^) compared to both LOW-GI (171 ± 41 mg·dL^−1^) and HIGH-GI (152 ± 28 mg·dL^−1^, *p* < 0.001). Additionally, LDL-C levels increased in LCHF (+17 ± 21 mg·dL^−1^, *p* = 0.001), while they decreased in both CHO groups (*p* < 0.05, respectively). Only the HIGH-GI group demonstrated a significant reduction in HDL-C (−3 ± 9 mg·dL^−1^, *p* = 0.006), while a decrease in TG was only significant in LOW-GI (−18 ± 36 mg·dL^−1^, *p* = 0.008).

**Conclusion:**

Although mean blood lipid levels remained within the normal range, the data indicate that a low-carbohydrate, high-fat (LCHF) diet leads to unfavorable changes in individual blood lipid profiles compared to carbohydrate-rich diets. Therefore, it is recommended that the impact of a low-carbohydrate diet on blood lipids be considered when counseling active and healthy individuals.

## 1 Introduction

In recent times, low-carbohydrate-high-fat (LCHF) diets have become a popular choice amongst endurance athletes seeking to enhance their capacity to utilize fat as a fuel source (1). A reduction in carbohydrate (CHO) intake results in a shift in substrate utilization toward a reliance on fat in circumstances where carbohydrate stores in the form of glycogen would typically be used (2–4). However, since carbohydrate stores are finite and a LCHF diet might promote increased fat oxidation while sparing CHO stores, this theory still appeals to some endurance athletes, despite official guidelines recommending a high-carbohydrate diet (5–8). In addition to the potential enhancements in their substrate metabolism, some athletes may also observe the favorable effects on weight and cardiometabolic health of a LCHF diet that have been evidenced in the general population. In this cohort, it has been demonstrated that an LCHF diet can have a beneficial impact on conditions such as obesity (9), metabolic syndrome (10) and type 2 diabetes (11). In light of these arguments, it appears reasonable to conclude that some endurance athletes view an LCHF diet as a healthy and effective approach.

In addition to the debate as to whether a LCHF should be recommended from a performance perspective (12–14), the cardiometabolic health benefits of a LCHF diet are frequently misinterpreted by endurance athletes in practice. Given that the majority of athletes already have a favorable health status, the interpretation of studies reporting health benefits of a LCHF diet must be approached with caution, as the reported improvements were often observed in individuals with overweight or existing metabolic disturbances. The assumption that an athlete’s physical condition and lifestyle are sufficient to protect them from cardiometabolic diseases, such as dyslipidemia, leads most athletes to be less concerned about the impact of their daily diet on their cardiometabolic health status. Instead, they focus on optimizing their daily intake of nutrients in order to enhance their training adaptations and competition performance.

Indeed, there is limited evidence, that a LCHF might lead to unfavorable alterations in blood lipid concentrations in endurance athletes (15). Athletes who follow a LCHF diet may experience an increase in total cholesterol (TC) levels. However, only three trials were included in the meta-analyses. Nevertheless, the findings should be regarded with respect by athletes who adhere to or plan to employ a LCHF diet, as they may already be at an elevated risk for arterial wall stiffening and myocardial fibrosis due to the high training volumes they engage in (16). In light of the ambiguous evidence regarding the performance benefits of a LCHF diet, endurance athletes who adhere to this dietary regimen may unintentionally elevate their risk of cardiovascular dysfunction. This could potentially negate the favorable cardiometabolic health outcomes achieved through training.

In addition, it still remains uncertain whether the findings on the impact of the glycemic index (GI) on blood lipids can be extrapolated to physically active individuals. Previous research has shown that reducing the GI can lead to beneficial changes in blood lipid levels in people with type 1 and 2 diabetes (17, 18) or obesity (19), as well as in young adults (20, 21). In fact, a meta-analysis of 28 randomized controlled trials in overweight and obese subjects has consistently shown that a diet containing low GI CHO can reduce TC and LDL-C. However, no effects on HDL-C or triglycerides (TG) were found (22). Compared to a LCHF diet, a low GI diet was associated with longer-lasting positive changes in cardiometabolic parameters, such as TC and HDL-C (19).

Despite the existing research on the impact of carbohydrate restriction on blood lipid levels in athletes (23–32), the current literature on this topic is still evolving and the acquisition of further data can contribute to a more nuanced understanding. Furthermore, the additional investigation of the GI should provide new insights into the effects of a high or low GI on blood lipids in athletic individuals. Thus, the main aim of this study was to investigate the impact of different nutritional regimens, which vary in carbohydrate content and GI, on blood lipid levels in recreationally active runners enrolled in a 10-week prescribed endurance training program. It was hypothesized that a LCHF diet would lead to higher levels of TC, LDL-C, and TG when compared to a carbohydrate-rich diet. Moreover, we considered that a low GI would have a beneficial impact on blood lipid profiles, in comparison to a high GI.

## 2 Materials and methods

This study is a secondary analysis from an investigation conducted at the University of Vienna (33). The original study followed an open, randomized, non-blinded design. The primary outcome variables in the present observation are distinct, with only the interventional data and subjects’ characteristics being shared. Accordingly, a comprehensive overview of the methodological approaches employed in the current study has been published elsewhere (33), but are summarized here for clarity. Specific methods only applied in this study will be provided in further detail. The registration of this study is located at ClinicalTrials.gov with the Identifier NCT05241730. This study adhered to all CONSORT guidelines (34). The original study protocol for this clinical trial, including the CONSORT diagram demonstrating participant flow, can be found in Moitzi, Krššák (33).

### 2.1 Participants

A summary of the subject’s characteristics can be found in Table 1. The initial cohort comprised 87 participants, who were randomly assigned to one of the three interventional groups. For various reasons, including non-compliance, infection with the SARS-CoV-2 virus, and personal withdrawal, 65 of the initial 87 recruited runners completed the study in its entirety.

**Table 1:** Subject characteristics.

	LOW-GI	HIGH-GI	LCHF	*p*-value
*N*	**24**	**20**	**21**	**–**
Age [years]	30 ± 4	29 ± 4	27 ± 4	0.112
Height [cm]	182 ± 7	180 ± 6	182 ± 7	0.740
Weight [kg]	79.5 ± 8.1	77.1 ± 11.3	81.5 ± 10.8	0.354
BMI [kg·m^−2^]	24.1 ± 2.8	23.7 ± 3.2	24.6 ± 3.3	0.661
Active days per week	3 ± 1	3 ± 1	3 ± 1	0.886
VO_2_ peak [mL·min^−1^·kg^−1^]	54 ± 7	55 ± 7	55 ± 9	0.967

The inclusion criteria required recreationally active (2–3 training sessions per week) male endurance athletes without any medical conditions. Exclusion criteria included experience within the last 6 months with one of the interventional diets, contraindication to physical activity according to the American College of Sports Medicine Guideline (35), use of medications or dietary supplements that could affect measurements or are prohibited by the WADA code, chronic diseases, and arterial hypertension.

The study protocol underwent review by the Ethical Committee of the Medical University of Vienna (EK Nr: 2105/2021), the Ethical Committee of the University of Vienna (Reference number: 00871), and was conducted in accordance with the declaration of Helsinki. Written informed consent was obtained from all participant prior to beginning of the intervention.

### 2.2 Intervention

The intervention lasted for a period of 10 weeks. Before the initial visit, all participants underwent a screening process that included a medical examination and an evaluation of their readiness for physical activity using the PAR-Q. Additionally, anthropometric and performance data, such as body composition and a graded exercise test, were collected; all of which are described in detail elsewhere (33). Enrolled participants were assigned to one of three groups (LOW-GI, HIGH-GI, and LCHF) based on their VO_2_ peak to minimize performance-related outcomes, as proposed by Hopkins (36).

Diet prescription is outlined in detail by Moitzi, Krššák (33). In brief, participants were instructed to adhere to their respective dietary patterns. All dietary regimens were designed as *ad libitum*, with subjects preparing their own meals in accordance with the respective group guidelines:

- LOW-GI: 50–60% carbohydrates with ≥65% of energy from low glycemic index (GI < 50) carbohydrates per day
- HIGH-GI: 50–60% carbohydrates with ≥65% of energy from high glycemic index (GI > 70) carbohydrates per day
- LCHF: ≥ 65% fat, maximum of 50 g carbohydrates per day.

The endurance exercise intervention was prescribed for all groups and consisted of five running sessions per week (three session constant moderate load, two sessions heavy strenuous load), with an average of 230 training minutes per week. The training zones were adjusted individually based on the results of the graded exercise test. The training was conducted individually by the test subjects, allowing for personal preferences regarding the time of day or the running route to be taken into account. The training sessions were uploaded onto the sports watch (Polar Vantage M, Polar Electro Oy, Kempele, Finnland) in advance, and during the session, the subjects received feedback from the watch via vibration indicating whether they were in the desired zone. Sessions were therefore recorded with a watch and a heart rate belt (Polar H10, Polar Electro Oy, Kempele, Finland) and controlled weekly by the study management. It was stated prior to the study that participants must complete a minimum of 75% of the prescribed training minutes to be included in the final analysis. Of the 87 participants, 11 were unable to achieve the requisite 75% of the prescribed training minutes.

### 2.3 Compliance evaluation

To monitor nutritional compliance, participants were instructed to record their food intake for one weekday and one weekend day per week. Trained dieticians reviewed the records using nut.s software (Dato Denkwerkzeuge, Wien, Austria). The food consumed was entered and analyzed in the software. In addition to the energy intake and macronutrient values, the fatty acids and fiber consumed were also analyzed in this study. Compliance during the study was assessed by calculating the mean of 20 24-h recalls per subject, which was then used for further calculations.

To assess nutrition prior to the intervention a 24-h recall and a food frequency questionnaire were used. The validated DEGS1-FFQ collects the frequency and quantity of 53 food items eaten in the last 4 weeks (37). The questionnaire was completed online and converted to nutritional intake according to previous proposed methods (38). For the baseline value the mean of the 24-h recall and the FFQ was used.

The determination of the GI of the diets was based on Atkinson, Brand-Miller (39) and Atkinson, Foster-Powell (40). To calculate the average GI of each recall, the percentage contribution of each individual CHO-containing food was multiplied by its glycemic index. The sum of these products was then divided by the number of meals and was taken as the GI of this recall. Finally, the mean GI of all protocols was determined for each subject in the carbohydrate groups. Given the markedly low carbohydrate intake observed in the LCHF cohort, the GI was not calculated for any of the participants in LCHF group.

### 2.4 Blood lipid biomarker analysis

Participants were instructed to arrive at the laboratory in the morning after an 8-h fasting period for the blood draw, both before and after the 10-week intervention. To minimize circadian influences blood samples were taken ±1 h before and after the intervention. A trained phlebotomist obtained an 8.5-ml blood sample through venipuncture. The serum sample (BD Vacutainer SST II Advance, Belliver Industrial Estate, Plymouth, United Kingdom) was sent to at a certified laboratory (Synlab, Institut für medizinische und chemische Labordiagnostik GmbH, Vienna, Austria) for subsequent analysis of total cholesterol (TC), HDL-C, LDL-C, and triglycerides (TG). There analyses for TC, HDL-C and TG were performed using an enzymatic color test in a clinical chemistry analyzer (AU5822, Beckman Coulter, Brea, USA). The respective Beckman Coulter kit numbers were OSR6216 for TC, OSR6287 for HDL-C, and ORS61118 for TG. LDL-C was calculated using the Friedewald equation, where:


$$LDL-C=TC-HDL-C-\frac{TG}{5}\text{.}$$

### 2.5 Statistical analyses

Statistical analyses were performed using the Statistical Package for the Social Sciences Software (SPSS for Windows, Version 28, SPSS Inc., Chicago, IL). Figures were created using GraphPad Prism (GraphPad Prism Version 8.0.2 for Windows, GraphPad Software, San Diego, California, United States). The level of significance was set at *α* = 0.05. Results are presented as mea*n* ± standard deviation (SD).

The normality of the distribution was evaluated using the Shapiro–Wilk test. The differences between groups at baseline and different changes during the intervention were evaluated using a one-way ANOVA and in case of no given normal distribution a Kruskal-Wallis test was used. If significant differences were identified, a Tukey post-hoc test was conducted to ascertain which groups exhibited significant differences. To assess differences in time (within-subject factor), group (between-subject factor), and time x group interaction effects, a two-way mixed ANOVA with Tukey-corrected *post hoc* analyses was conducted. In the event of a significant interaction, simple main effects for group and time were analyzed. For significant results, we displayed effect sizes for one-way ANOVA (ηp^2^) and simple time effects (Cohen’s *d*). Due to observed baseline differences in LDL-C levels among the groups, a one-way ANCOVA was employed to adjust for these initial disparities and assess the effect of the interventions on follow-up LDL-C levels. Baseline LDL-C level was included as a covariate to control for initial differences among participants. *Post hoc* comparisons were performed using the Bonferroni correction to adjust for multiple testing.

In order to ascertain the relationship between alterations in blood lipid levels, body weight and dietary intake, a Pearson’s correlation was conducted. If normal distribution was not given, Spearman was used. Therefore, the change was calculated as value post minus value pre (*Δ*). Variables used for the correlation analysis included TC, HDL-C, LDL-C, TG, body weight, energy intake, relative macronutrient intake, glycemic index, fatty acids intake and fiber intake.

Lastly, a multiple linear regression was conducted to refine predictive models for changes in blood lipid concentrations (ΔTC, ΔHDL-C, ΔLDL-C and ΔTG), initially including changes in energy intake, relative macronutrient intake (CHO, proteins, fat), glycemic index, fiber intake, body weight and composition (fat mass, fat-free mass) as potential predictors. The analysis utilized a backward elimination method, with a criterion set at a probability of F-to-remove greater than or equal to 0.100. The predictors with the highest adjusted R^2^ from the backward elimination method were included in the final model. A multiple linear regression was calculated with those predictors using the enter method. Linearity was assessed by visual interpretation of partial regression plots and a plot of studentized residuals against the predicted values. Homoscedasticity was assessed via visual inspection of a plot of studentized residuals versus unstandardized predicted values. When independent variables had a correlation coefficient *R* > 0.8 and the multicollinearity was harmed, one of the parameters was removed from the model.

Eight participants lacked baseline data on dietary fiber and fatty acid intake due to issues with data collection. Nevertheless, the energy intake and macronutrient intake of these participants were evaluated using the FFQ.

## 3 Results

### 3.1 Study population

As already stated detailed information about subjects, body weight and composition and performance-measurements are published in Moitzi, Krššák (33). For clarity, subjects baseline characteristics are shown in Table 1.

### 3.2 Nutritional intervention

Detailed nutritional data are already published in Moitzi, Krššák (33). Data on energy intake and relative macronutrient intake will be presented in brief in the following. Energy intake was significantly reduced in LOW-GI (T-0: 2178 ± 556 kcal vs. T-10: 1784 ± 502 kcal, *p* < 0.001, *d* = 0.802). During the intervention, intake was significantly higher in HIGH-GI (2124 ± 462 kcal) compared to the LCHF (1755 ± 468 kcal, *p* = 0.043, ηp^2^ = 0.109). Analysis revealed significant interaction effects for relative carbohydrate, protein, and fat intake. During the intervention, LOW-GI (50.5 ± 5.4%) and HIGH-GI (53.5 ± 5.6%) had a significantly higher relative carbohydrate intake compared to LCHF (10.6 ± 3.7%, *p* < 0.001, ηp^2^ = 0.941). All groups experienced a significant change in relative protein intake. LOW-GI (+3.4 ± 2.4%, *p* < 0.001, *d* = 1.452) and LCHF (+9.3 ± 4.7%, *p* < 0.001, *d* = 1.975) had an increase in relative protein intake, while HIGH-GI (−1.1 ± 2.3%, *p* = 0.042, *d* = 0.487) had a decrease. There were significant differences in protein intake between all groups (for all pairwise comparisons *p* < 0.050, ηp^2^ = 0.773). The LOW-GI group experienced a decrease in relative fat intake (−3.1 ± 6.9%, *p* = 0.034, *d* = 0.461), while the LCHF group experienced an increase (+27.9 ± 8.9%, *p* < 0.001, *d* = 3.152) and intake in HIGH-GI remained unchanged (−2.0 ± 5.2%, *p* = 0.104). Fat intake also differed significantly between the LCHF group and the two carbohydrate groups (*p* < 0.001, ηp^2^ = 0.906). The study evaluated the glycemic index for LOW-GI and HIGH-GI. The study found that the glycemic index decreased in the LOW-GI group (−22 ± 9, *p* < 0.001, *d* = 1.469) and increased in the HIGH-GI group (+7 ± 7, *p* < 0.001, *d* = 0.993). There was a significant difference in the glycemic index between the two groups during the intervention (LOW-GI: 41 ± 3, HIGH-GI: 64 ± 3, *p* < 0.001, *d* = 7.555).

In addition to macronutrient intake, the intake of saturated fatty acids (SFA), monounsaturated fatty acids (MUFA), polyunsaturated fatty acids (PUFA), omega-3 (n-3-FA) and omega-6 fatty acids (n-6-FA), total fiber, soluble and insoluble fiber were determined from the food protocols and are shown in Table 2. SFA intake was significantly reduced in LOW-GI (−7.7 ± 15.4 g·day^−1^, *p* = 0.025, *d* = 0.529), while in LCHF, SFA intake increased (+34.3 ± 16.6 g·day^−1^, *p* < 0.001, *d* = 2.065). Intake during the study was significantly higher in the LCHF group compared to the LOW-GI or HIGH-GI group (*p* < 0.001, ηp^2^ = 0.458). Intake of MUFA showed no difference between the LOW-GI and HIGH-GI group, whereas intake in the LCHF group increased significantly (+31.6 ± 30.5 g·day^−1^, *p* < 0.001, *d* = 1.036) and was significantly higher during the study compared to both other groups (*p* < 0.001, ηp^2^ = 0.513). Additionally, n-3-FA intake increased in LCHF (+3.4 ± 4.0 g·day^−1^, *p* = 0.004, *d* = 0.860) with a significantly higher intake during the intervention compared to LOW-GI or HIGH-GI (*p* < 0.001, ηp^2^ = 0.454). Intake of PUFA or n-6-FA showed no significant interaction effect. However, the main effect for the group showed a significantly higher mean PUFA-intake in LCHF (24.2 ± 13.7 g·day^−1^) compared to LOW-GI (16.0 ± 6.0 g·day^−1^, *p* = 0.008, ηp^2^ = 0.163) independent of time.

**Table 2:** Fatty acids and fiber intake before (T-0) and during the intervention.

	Group	T-0	During the intervention (T-10)	Time x Group	Simple group effect at T-10
SFA [g·day^−1^]	LOW-GI	35.9 ± 16.6	26.9 ± 13.2 ^c,*^	**<0.001**	**<0.001**
	HIGH-GI	31.9 ± 20.3	36.0 ± 14.2 ^c^		
	LCHF	30.4 ± 16.3	60.4 ± 19.7 ^a,b,*^		
MUFA [g·day^−1^]	LOW-GI	30.0 ± 12.8	27.9 ± 10.6 ^c^	**<0.001**	**<0.001**
	HIGH-GI	34.0 ± 21.1	31.6 ± 11.5 ^c^		
	LCHF	36.2 ± 24.6	63.2 ± 22.7 ^a,b,*^		
PUFA [g·day^−1^]	LOW-GI	16.0 ± 7.2	16.0 ± 5.1	0.135	**0.008 # (LCHF vs. LOW-GI)**
	HIGH-GI	19.8 ± 11.8	18.5 ± 6.0		
	LCHF	20.5 ± 15.6	27.0 ± 12.2		
N-3-FA [g·day^−1^]	LOW-GI	2.1 ± 1.5	2.3 ± 0.7 ^c^	**<0.001**	**<0.001**
	HIGH-GI	4.7 ± 6.0	3.3 ± 2.1 ^c^		
	LCHF	3.2 ± 2.0	6.5 ± 2.8 ^a,b,*^		
N-6-FA [g·day^−1^]	LOW-GI	13.9 ± 7.1	13.7 ± 4.6	0.473	0.073 #
	HIGH-GI	15.9 ± 11.1	15.2 ± 5.5		
	LCHF	17.4 ± 14.6	20.5 ± 10.0		
Fiber [g·day^−1^]	LOW-GI	35.2 ± 12.9	40.8 ± 8.9 ^b, c^	**0.038**	**<0.001**
	HIGH-GI	32.9 ± 8.6	28.0 ± 8.8 ^a^		
	LCHF	28.2 ± 13.9	25.9 ± 9.4 ^a^		
Soluble fiber [g·day^−1^]	LOW-GI	10.7 ± 4.0	12.1 ± 2.8 ^c^	**0.014**	**<0.001**
	HIGH-GI	11.6 ± 3.2	10.4 ± 4.4 ^c^		
	LCHF	8.4 ± 4.5	5.9 ± 2.4 ^a,b,*^		
Insoluble fiber [g·day^−1^]	LOW-GI	24.1 ± 9.4	28.7 ± 6.3	0.068	**<0.001 # (LCHF vs. LOW-GI)**
	HIGH-GI	21.3 ± 7.7	19.0 ± 5.2		
	LCHF	19.1 ± 10.4	18.1 ± 7.6		

^a,b,c^ Significantly different intake between groups during the intervention: ^a^compared to LOW-GI, ^b^compared to HIGH-GI, ^c^compared to LCHF. T-0: baseline values, T-10: values during the intervention. * significant different from T-0. # reflects *p*-value of main effects. Bold numbers represent a significant *p*-value.

The fiber intake during the study was significantly higher in LOW-GI compared to LCHF or HIGH-GI (*p* < 0.001, ηp^2^ = 0.369). Intake of soluble fiber decreased in LCHF (−2.9 ± 4.7 g·day^−1^, *p* = 0.026, *d* = 0.619) and differed significantly from LOW-GI and HIGH-GI during the study (*p* < 0.001, ηp^2^ = 0.407). The LOW-GI group showed a significantly higher mean intake in insoluble fiber compared to LCHF group (*p* < 0.001, ηp^2^ = 0.307) independent of time.

### 3.3 Exercise intervention

The training minutes were divided into basic and interval sessions, and no significant difference was found between the groups (*p* > 0.05). Additionally, there were no differences in total training minutes between the LOW-GI (2,125 ± 294 min), HIGH-GI (2072 ± 285 min), and LCHF (2,103 ± 256 min) groups (*p* = 0.824).

### 3.4 Blood lipid levels

**Figure 1:**
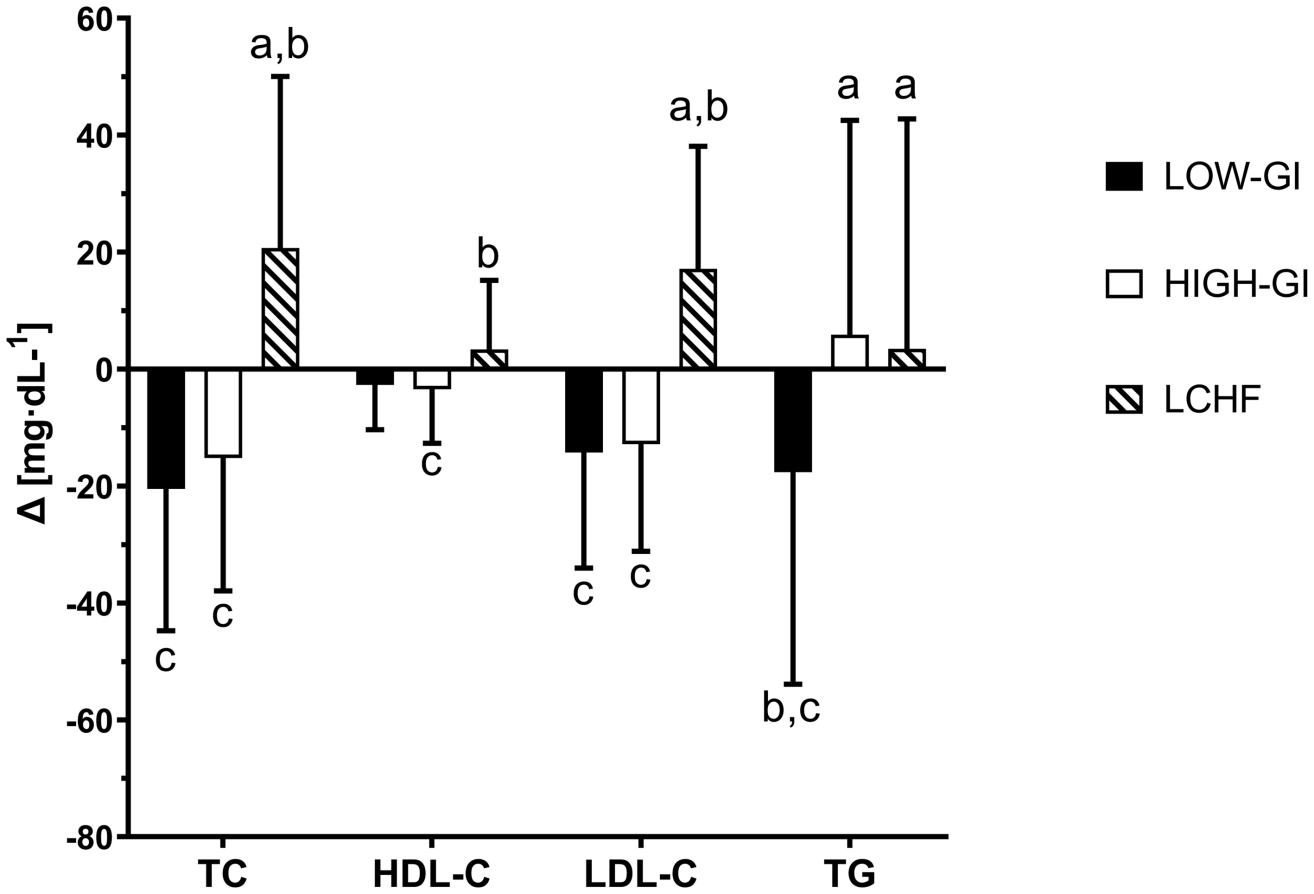
Different changes in blood lipid levels during 10-week intervention: a compared to LOW-GI, b compared to HIGH-GI, c compared to LCHF. TC, total cholesterol; TG, triglyzerides.

At baseline measurement, blood lipid levels did not differ except for LDL-C. LDL-C was significantly higher in LOW-GI compared to HIGH-GI (*p* = 0.035, ηp^2^ = 0.095). Two-way mixed ANOVA showed significant time group interactions for TC, HDL-C and LDL-C (*p* < 0.05, respectively, see Table 3). The study found that TC levels significantly decreased in both LOW-GI (−21 ± 24 mg·dL^−1^, *p* < 0.001, *d* = 0.845) and HIGH-GI (−15 ± 23 mg·dL^−1^, *p* = 0.007, *d* = 0.669), while it increased in LCHF (+21 ± 29 mg·dL^−1^, *p* = 0.004, *d* = 0.706). After the intervention, TC was significantly higher in LCHF compared to LOW-GI or HIGH-GI (*p* < 0.001, ηp^2^ = 0.201) and increase was significantly greater in LCHF (*p* < 0.001, ηp^2^ = 0.589). There was a significant decrease in HDL-C in HIGH-GI (−3 ± 9 mg·dL^−1^, *p* = 0.006, *d* = 0.374), while no changes were observed in LOW-GI or LCHF. Changes in HDL-C were significantly different between HIGH-GI and LCHF (*p* = 0.043, ηp^2^ = 0.098). At T-10, there were no differences in HDL-C. LDL-C decreased significantly in LOW-GI (−14 ± 20 mg·dL^−1^, *p* = 0.002, *d* = 0.723) and HIGH-GI (−13 ± 18 mg·dL^−1^, *p* = 0.005, *d* = 0.702) and increased in LCHF (+17 ± 21 mg·dL^−1^, *p* = 0.001, *d* = 0.818). The change in LDL-C was significantly higher in LOW-GI and HIGH-GI compared to LCHF (*p* < 0.001, ηp^2^ = 0.359). Due to baseline differences in LDL-C concentration a one-way ANCOVA was employed to compare groups at T-10. Prior to conducting the ANCOVA, assumptions were assessed. Linearity between the baseline LDL-C and follow-up LDL-C was confirmed using scatterplots within each group. The homogeneity of regression slopes assumption was tested by examining the interaction between the group and baseline LDL-C levels, which was not statistically significant [F (2, 29) = 1.798, *p* = 0.175], indicating that the assumption was met. Normality of residuals was evaluated using Q-Q plots and the Shapiro–Wilk test, confirming normal distribution (*p* > 0.05). Homogeneity of variances was assessed using Levene’s Test of Equality of Error Variances, which was not significant [F(2, 62) = 2.054, *p* = 0.137]. After adjustment for pre-intervention LDL-C concentration, there was a statistically significant difference in LDL-C concentration at T-10 [F(2, 61) = 20.391, *p* < 0.001, ηp^2^ = 0.401]. Adjusted LDL-C concentration at T-10 was statistically significantly greater in LCHF (*M* = 115, SE = 4 mg·dL^−1^) compared to LOW-GI (*M* = 87, SE = 4 mg·dL^−1^, *p* < 0.001) and HIGH-GI (*M* = 82, SE = 4 mg·dL^−1^, *p* < 0.001). TG showed no significant interaction effect. However, one way ANOVA of *Δ*-values revealed a significant higher decrease in LOW-GI compared to HIGH-GI or LCHF (*p* = 0.009, ηp^2^ = 0.149, Figure 1).

**Table 3:** Blood lipid levels at baseline (T-0) and after the intervention (T-10).

	Group	T-0	T-10	Time x Group	Simple group effect at T-10
TC [mg·dL^−1^]	LOW-GI	191 ± 43	171 ± 41 ^*,c^	**<0.001**	**<0.001**
	HIGH-GI	167 ± 37	152 ± 28 ^*,c^		
	LCHF	175 ± 33	196 ± 37 ^*,a,b^		
HDL-C [mg·dL^−1^]	LOW-GI	61 ± 13	59 ± 11	**0.048**	0.197
	HIGH-GI	61 ± 12	57 ± 11 ^*^		
	LCHF	60 ± 14	64 ± 13		
LDL-C [mg·dL^−1^]	LOW-GI	109 ± 32 ^b^	95 ± 34 ^*,b,c^ *87 ± 4 ^c^*	**<0.001**	***<0.001***
	HIGH-GI	86 ± 32 ^a^	73 ± 21 ^*,a,c^ *82 ± 4 ^c^*		
	LCHF	98 ± 28	115 ± 30 ^*,a,b^ *115 ± 4 ^a,b^*		
TG [mg·dL^−1^]	LOW-GI	102 ± 49	85 ± 36	0.074	0.574^#^
	HIGH-GI	100 ± 74	106 ± 75		
	LCHF	85 ± 38	89 ± 36		

^a,b,c^ significantly different concentration between groups after the intervention: ^a^compared to LOW-GI, ^b^compared to HIGH-GI, ^c^compared to LCHF. *significant different from T-0. ^#^reflects *p*-value of main effects. Bold numbers represent a significant *p*-value. Italic numbers represent estimates ± standard error and results of ANCOVA analysis.

### 3.5 Correlation between changes in blood lipid levels, body weight, and nutrition

**Figure 2:**
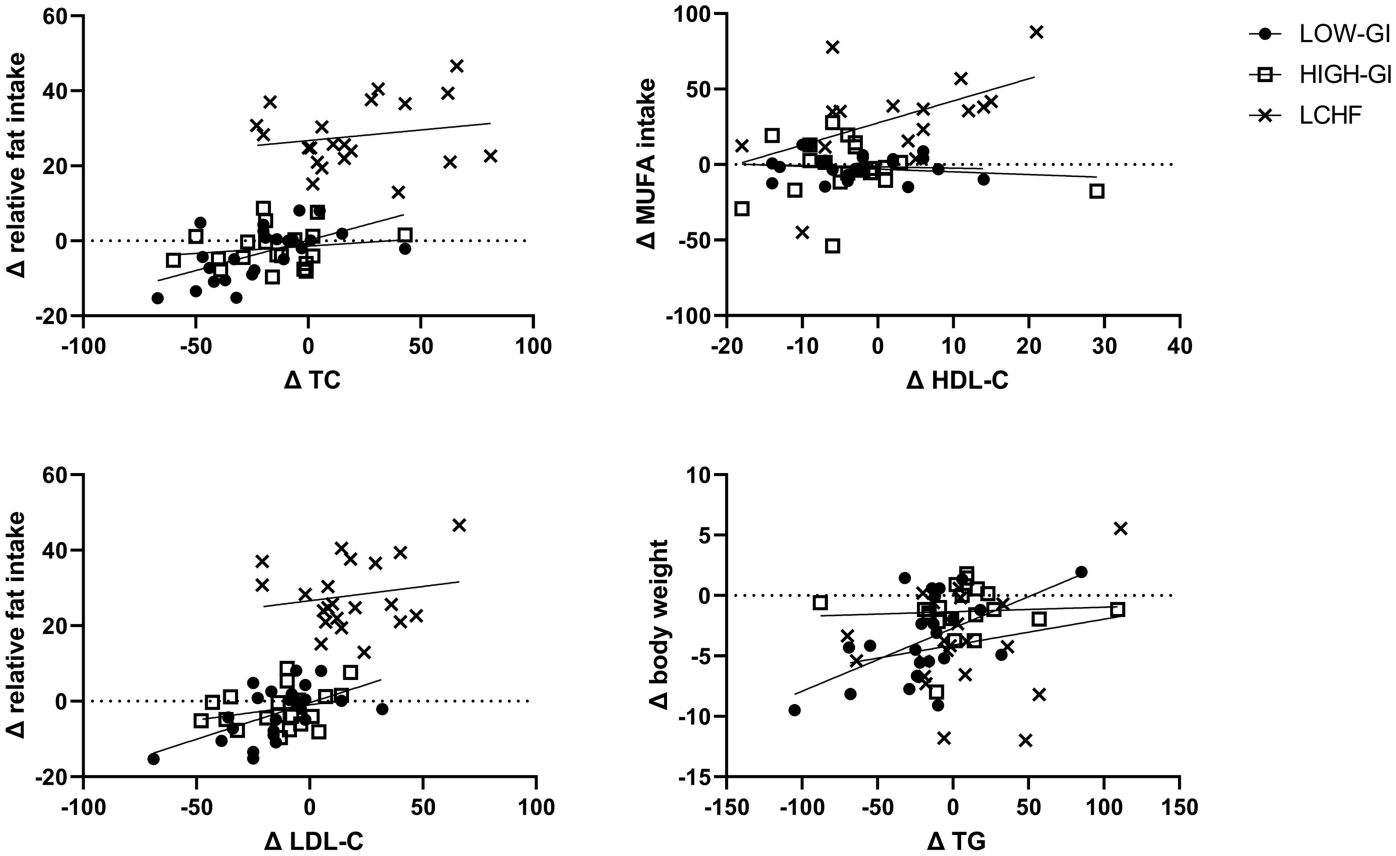
Significant correlations between changes in blood lipids and dietary intake or body weight. TC, total cholesterol; MUFA, monounsaturated fatty acids; TG, triglyzerides.

Changes in TC were significantly correlated with changes in relative protein intake (*p* = 0.017, *r* = 0.295), relative CHO intake (*p* < 0.001, *r* = −0.580), relative fat intake (*p* < 0.001, *r* = 0.639), SFA intake (*p* < 0.001, *r* = 0.461), MUFA intake (*p* < 0.001, *r* = 0.494) and n-3-FA intake (*p* = 0.048, *r* = 0.264). Changes in relative protein intake (*p* = 0.043, *r* = 0.252), relative CHO intake (*p* = 0.009, *r* = −0.323), relative fat intake (*p* = 0.008, *r* = 0.328) and MUFA intake (*p* = 0.012, *r* = 0.332) were significantly correlated with changes in HDL-C. Changes in LDL-C were significantly correlated with changes in relative protein intake (*p* = 0.014, *r* = 0.305), relative CHO intake (*p* < 0.001, *r* = −0.588), relative fat intake (*p* < 0.001, *r* = 0.655), SFA intake (*p* < 0.001, *r* = 0.502), MUFA intake (*p* < 0.001, *r* = 0.487) and n-3-FA intake (*p* = 0.006, *r* = 0.360). Only changes in body weight (*p* = 0.011, *r* = 0.314) were correlated with changes in TG. All other comparisons yielded insignificant correlations. Highest correlation of TC, HDL-C, LDL-C and TG with respective parameters are shown in Figure 2.

### 3.6 Multiple linear regression

The backward elimination method was employed to ascertain the most appropriate predictor variables for *Δ* total cholesterol. The results indicated that the predictor variables ΔBMI, *Δ* relative fat intake and *Δ* energy intake exhibited the highest adjusted *R*^2^ of 0.210. The multiple regression model statistically significantly predicted ΔTC [F(3, 61) = 20.158, *p* < 0.001]. *R*^2^ for the model was 49.8% with an adjusted *R*^2^ of 47.3%. *Δ* BMI and Δ relative fat intake added statistically significantly to the prediction (*p* < 0.05). Regression coefficients and standard errors can be found in Table 4.

**Table 4:** Multiple regression results for Δ total cholesterol concentration.

Total cholesterol	*B*	95% CI for B		SE B	Beta	*R* ^2^	Δ*R*^2^
		LL	UL				
Model						0.50	0.47
Constant	−6.35	−14.39	1.68	4.02			
ΔBMI [kg·m−2]	8.98**	3.38	14.58	2.80	0.30		
ΔEnergy intake [kcal]	0.00	−0.01	0.01	0.01	0.01		
ΔRelative fat Intake [% of total EI]	1.30***	0.94	1.65	0.18	0.67		

Model, “enter” method in SPSS statistics; EI, energy intake; *B*, unstandardized regression coefficient; CI, confidence interval; LL, lower limit; UL, upper limit; SEB, standard error of the coefficient; beta, standardized coefficient; *R*^2^, coefficient of determination; *ΔR*^2^, adjusted *R*^2^; **p < 0.01; ***p < 0.001.

For the *Δ*HDL-C concentration most appropriate variables according to backward elimination method are Δ fiber intake, Δ BMI, Δ relative fat intake, Δ relative CHO intake and Δ energy intake (adjusted *R*^2^ = 0.097). Δ relative CHO intake was removed from the final model due to a R greater 0.8. *R*^2^ for the final model was 20.0% with an adjusted *R*^2^ of 13.9%. The parameters statistically significantly predicted *Δ*HDL-C [F(4, 52) = 3.253, *p* = 0.019]. *Δ* BMI and Δ relative fat intake added statistically significantly to the prediction (*p* < 0.05). Results are presented in Table 5.

**Table 5:** Multiple regression results for ΔHDL-C concentration.

HDL-C	*B*	95% CI for B		SE B	Beta	*R* ^2^	Δ*R*^2^
		LL	UL				
Model						0.20	0.14
Constant	−1.43	−4.57	1.72	1.57			
ΔBMI [kg·m−2]	2.54*	0.21	4.87	1.16	0.29		
ΔEnergy intake [kcal]	0.00	−0.01	0.00	0.00	−0.20		
ΔRelative Fat Intake [% of total EI]	0.24**	0.09	0.39	0.08	0.40		
ΔFiber intake [g·day−1]	0.06	−0.13	0.25	0.09	0.09		

Model, “enter” method in SPSS statistics; EI, energy intake; *B*, unstandardized regression coefficient; CI, confidence interval; LL, lower limit; UL, upper limit; SEB, standard error of the coefficient; beta, standardized coefficient; *R*^2^, coefficient of determination; Δ*R*^2^, adjusted *R*^2^; *p < 0.05; ** *p* < 0.01.

Backward elimination method revealed *Δ* BMI, Δ fat-free mass, Δ fat mass, Δ fiber intake, Δ energy intake and Δ relative fat and CHO intake to be the most appropriate predictors for ΔLDL-C concentration (adjusted *R*^2^ = 0.123). The final model was constructed without *Δ* relative CHO intake, Δ fat-free mass and Δ fat mass, as *R* > 0.8 or redundant information was present, which resulted in a decrease in adjusted *R*^2^. The multiple regression model statistically significantly predicted ΔLDL-C concentration [F(4, 52) = 10.756, *p* < 0.001, R^2^ = 0.453, adjusted *R*^2^ = 0.411]. Δ BMI and Δ relative fat intake added statistically significantly to the prediction (*p* < 0.05, Table 6).

**Table 6:** Multiple regression results for ΔLDL-C concentration.

LDL-C	*B*	95% CI for B		SE B	Beta	*R* ^2^	Δ*R*^2^
		LL	UL				
Model						0.45	0.41
Constant	−6.29	−12.62	0.03	3.15			
ΔBMI [kg·m^−2^]	5.55*	0.87	10.23	2.33	0.26		
ΔEnergy intake [kcal]	0.00	−0.02	0.01	0.01	−0.10		
ΔRelative fat intake [% of total EI]	0.97***	0.67	1.28	0.15	0.67		
ΔFiber intake [g·day^−1^]	0.25	−0.13	0.63	0.19	0.15		

Model, “enter” method in SPSS statistics; EI, energy intake; *B*, unstandardized regression coefficient; CI, confidence interval; LL, lower limit; UL, upper limit; SEB, standard error of the coefficient; *R*^2^ = coefficient of determination; Δ*R*^2^ = adjusted *R*^2^; *p < 0.05, ***p < 0.001.

The backward method revealed that the following variables were suited predictors of ΔTG: Δ BMI, Δ fat-free mass, Δ fat mass, Δ energy intake, Δ relative CHO and fat intake and Δ fiber intake (adjusted *R*^2^ = 0.357). However, the final model was constructed without the inclusion of Δ CHO intake and Δ fat mass (*R* > 0.8). The remaining predictors were found to significantly predict ΔTG [F(5, 51) = 3.772, *p* = 0.006, *R*^2^ = 0.270, adjusted *R*^2^ = 0.198]. Δ energy intake and Δ fiber intake were identified as significant contributors to the prediction (*p* < 0.05, Table 7).

**Table 7:** Multiple regression results for ΔTG concentration.

Triglycerides	*B*	95% CI for B		SE B	Beta	*R* ^2^	Δ*R*^2^
		LL	UL				
Model						0.27	0.20
Constant	7.85	−5.77	21.47	6.78			
ΔBMI [kg·m^−2^]	11.47	−0.19	23.13	5.81	0.30		
ΔFat-free mass [kg]	−1.05	−8.71	6.62	3.82	−0.04		
ΔEnergy intake [kcal]	0.02*	0.00	0.05	0.01	0.28		
ΔRelative Fat Intake [% of total EI]	0.47	−0.17	1.12	0.32	0.19		
ΔFiber intake [g·day-1]	−1.00*	−1.78	−0.22	0.39	−0.34		

Model, “enter” method in SPSS statistics; EI, energy intake; *B*, unstandardized regression coefficient; CI, confidence interval; LL, lower limit; UL, upper limit; SEB, standard error of the coefficient; beta, standardized coefficient; *R*^2^, coefficient of determination; Δ*R*^2^, adjusted *R*^2^; *p < 0.05.

## 4 Discussion

The data presented indicate that a 10-week LCHF diet may result in unfavorable changes in blood lipid panel when compared to a carbohydrate-rich diet in recreationally active runners. In particular, the data show increased levels of TC and LDL-C after the LCHF diet, whereas TC and LDL-C decreased during the carbohydrate rich diets. Additionally, a significant reduction in HDL-C was observed in the HIGH-GI group.

Previous studies have similarly demonstrated the onset of chronic hypercholesterolemia following the adoption of a low-carbohydrate, high-fat (LCHF) diet (25, 26, 28, 31, 32). The notable elevation in TC in the present observation may be attributed to the considerable rise in LDL-C, while no alterations were discerned in HDL-C following the LCHF diet. This differs from other studies, in which an increase in TC was accompanied by an increase in both HDL-C and LDL-C (26, 31) or HDL-C alone (25, 28). Additionally, in contrast to previous observations (24, 25, 30, 31), a LCHF diet was not found to have a “triglyceride-lowering” effect. It is possible that discrepancies may arise in the outcomes due to variations in the methodological approaches (*ad libitum*, isoenergetic diets or free-living), the different types of training (aerobic or anaerobic activities) and the populations (endurance or power athletes) studied. To elucidate the presented outcomes, we propose several potential explanations.

Compared with normative thresholds for dyslipidemia and cardiovascular risk (41), the lipid panel of the CHO-rich diets was in range. For the LCHF group, only the LDL-C concentration was above optimal (LDL-C: 115 mg·dL^−1^) with the TC concentration still being in range. The increases in TC and LDL-C following an LCHF diet were anticipated for several reasons. Diets were designed *ad libitum* without any restrictions regarding the fatty acid intake. In general, it is recommended that a LCHF diet should have a higher intake of monounsaturated (MUFA) and polyunsaturated (PUFA) fatty acids, while limiting the intake of saturated fatty acids (SFA) (42, 43). During the *ad libitum*, non-restrictive LCHF diet, our group of recreationally active men experienced a significant change in dietary fat intake to meet energy needs. While the intake of SFA decreased in LOW-GI and remained unchanged in HIGH-GI, it increased by around 50% in LCHF compared to baseline intake. Furthermore, a moderate positive correlation was observed between changes in SFA intake and TC and LDL-C. This suggests that higher SFA intake may stimulate cholesterol biosynthesis, leading to increased circulating cholesterol (44). It is important to note that an optimal intake of SFA, MUFA, and PUFA can significantly influence serum cholesterol and lipoprotein levels (45). However, it was observed that in the LCHF group, the higher intake of MUFA and n-3-FA did not lead to favorable changes in TC, lipoprotein levels, or TG. This may be partly explained by the concurrent higher intake of SFA, as the observed correlation between SFA intake and TC or LDL-C was stronger compared to the correlation between n-3-FA and TC or LDL-C. On the other hand, low GI nutrition led to a decrease in SFA intake, accompanied by a decrease in TC and LDL-C. No significant changes in fatty acid intake were observed in the HIGH-GI group. However, it could be speculated that, endurance exercise may have resulted in a decrease in TC and LDL-C levels.

It is clear that regular moderate endurance exercise of around 150 min or 75 min of vigorous exercise per week provides reliable protection against cardiovascular disease (46). Our subjects increased their endurance exercise from an average of three sessions per week to five sessions per week during the intervention, and there was no difference between the groups. Aerobic exercise seems to have a greater impact on HDL-C levels compared to LDL-C or TG. This is because aerobic exercise increases the concentration and activity of lipoprotein lipase in skeletal muscles, which in turn increases HDL-C levels (47). However, the improved function of HDL-C, specifically in terms of increased reverse cholesterol transport and lipid peroxide transport clearing, requires further investigation (48). The effects of aerobic exercise on LDL-C are still unclear and require more data. Aerobic exercise might reduce smaller and less dense LDL-subfractions, which are directly linked to cardiovascular events (49). Additionally, exercise can lead to lower TG concentrations, because it appears that there is an inverse relationship between HDL-C and TG (50). It is unclear to what extend the endurance exercise program had an impact on our results, because subjects were already moderately trained before enrolment. However, it seems that the higher dietary fat intake in the LCHF group contributed greater to alterations in blood lipids compared to the regular endurance exercise.

Weight loss and energy intake has a large beneficial effect on circulating blood lipids, making it difficult to separate dietary effects from other factors (51, 52). Particularly in this case, subjects in all groups experienced significant reductions in body weight, BMI and absolute fat mass [data presented in Graybeal et al. (33)], with significantly greater losses in the LOW-GI and LCHF groups. However, as both groups (LCHF and LOW-GI) experienced similar weight loss, the increase in TC and LDL-C may be partly due to the significantly higher fat intake in LCHF compared to LOW-GI (53). Furthermore, energy intake was markedly diminished in the LOW-GI and LCHF groups, exhibiting lower energy intake than the HIGH-GI group. It can thus be concluded that energy intake alone is not responsible for changes in the blood lipid panel.

Furthermore, we found some additional effects of glycemic index on TC or subfractions. The low GI diet resulted in significantly higher reductions in TG compared to the high GI and the LCHF diet. Epidemiological evidence suggests an inverse correlation between GI and HDL-C (54, 55) and a positive effect on total and LDL cholesterol (22, 56). The higher GI in the HIGH-GI group might therefore be responsible for the significant decrease in HDL-C after 10 weeks. A similar decline has been documented in previous studies (21, 57–59). Nevertheless, the underlying mechanism remains to be elucidated. Chronic hyperinsulinemia might stimulate a series of interconnected metabolic processes (60), including for example an increased production of very-low-density lipoproteins via the upregulation of cholesteryl ester transport protein (61, 62), altered HDL-C composition (63), or reduced cholesterol efflux (64) thereby reducing reverse cholesterol transport (65, 66). Moreover, it appears that a combination of vascular dysfunction and endothelial damage, which has also been observed during chronic hyperinsulinemia, may be responsible for the reduction in HDL-C levels following a HIGH-GI diet (58). Additionally, dietary fiber and GI work together to affect lipid absorption and synthesis. Sources rich in insoluble fiber appear to have a smaller effect on serum lipids compared to sources rich in soluble fiber, which have been shown to effectively lower lipids (53, 67). In this investigation, the intake of soluble fiber was significantly higher in the LOW-GI group compared to the HIGH-GI or LCHF groups, resulting in the greatest decrease in TC (not significant compared to HIGH-GI), LDL-C (not significant compared to HIGH-GI) and TG in the LOW-GI group. However, no correlation was found between the change in fiber intake and the changes in blood lipids. The responsible mechanisms still need to be addressed, but two main factors have been proposed to influence the decrease in blood lipids after fiber intake. Firstly, increased dietary fiber intake leads to reductions in bile acid and cholesterol absorption from the ileum, which inhibits hepatic cholesterol synthesis. Secondly, a low GI diet can lead to reduced insulin secretion, which in turn reduces the activity of hydroxy-3-methylglutaryl-CoA reductase, the rate-limiting enzyme of cholesterol synthesis (44). Nevertheless, according to this explanation, the LCHF group with reduced insulin production should also experience favorable changes in the lipid profile. Therefore, more data are needed to understand the underlying mechanism in active individuals.

Finally, additional data are required to ensure the accurate prediction of changes blood lipid levels based on dietary intake, body weight, and composition. The adjusted R^2^ for the calculated models ranged from 14 to 47%. Examining the relationship between change in TC reveals that relative fat intake and BMI serve as reliable predictors. Positive correlations were observed between changes in BMI and relative fat intake. Specifically, an increase in BMI of 1 kg·m^−2^ is associated with a 9 mg·dL^−1^ rise in TC, which is consistent with the findings of previous studies in patients with diabetes (68) and obese individuals (69). Additionally, a 10% increase in relative fat intake is associated with a 13 mg·dL^−1^ rise in TC. These findings contrast with previous studies on overweight individuals (70, 71), which recommend an increased fat intake for weight loss and improved lipid profiles. However, research on athletic populations indicates that the results may not be directly applicable to recreationally active subjects (25, 26, 28, 31, 32). This data suggests that an increase in relative fat intake may contribute to the development of hypercholesterolemia. The predictors for changes in HDL-C exhibited low reliability, with an adjusted R^2^ of only 14%. The anticipated relationship between BMI and relative fat intake was not observed. The model suggests that an increase in BMI of 1 kg·m^−2^ and 10% increase in relative fat intake are associated with increases in HDL-C of 3 mg·dL^−1^ and 2 mg·dL^−1^, respectively. Further data are required to substantiate these findings (72). A positive correlation was identified between changes in BMI and relative fat intake and changes in LDL-C. An increase in BMI by 1 kg·m^−2^ was associated with a 6 mg·dL^−1^ increase in LDL-C, and a 10% increase in relative fat intake resulted in an increase of 10 mg·dL^−1^ in LDL-C. These findings differ from results in obese subjects (70, 71, 73), but align with previous reports in athletes (26, 31) or normal-weight adults (72). Dietary fiber and energy intake were reliable predictors of changes in TG levels. A 1000 kcal increase in energy intake resulted in a 23 mg·dL^−1^ increase in TG, a finding consistent with previous studies (51). Additionally, changes in fiber intake and TG were inversely correlated, with a 10-gram increase in daily fiber intake leading to a 10 mg·dL^−1^ reduction in TG. This observation warrants further analysis, as it has yet to be empirically validated (74, 75). Therefore, further investigation is required to ascertain the reliability of regression models in predicting changes in blood lipid concentrations based on change in nutrient intake and body weight in healthy, active individuals. The lack of a sufficiently large sample size precludes any definitive conclusions. Nevertheless, the results suggest that, in addition to BMI, fat intake significantly influences blood lipids. Therefore, individuals who are regularly physically active and considering a LCHF diet should carefully evaluate its potential effects on their blood lipid profile. Existing literature on obese patients cannot be extrapolated to this population.

### 4.1 Limitations

In addition to the study’s strengths, such as the intervention’s duration and free-living conditions, it is important to mention some limitations. The diet’s compliance was evaluated through 24-h recalls for 2 days per week, which may have caused distortions. Additionally, it is important to note that this trial only included male athletes. It remains unclear whether sex has an impact on the response of blood lipid levels to diet and exercise. Moreover, it is currently not possible to ascertain from the available data how long the effects of a LCHF diet on blood lipids will persist. It is unclear how long it will take for blood lipid levels to return to their original baseline levels following a transition to the habitual diet.

## 5 Conclusion

A LCHF diet is often recommended for weight loss and fat oxidation in active individuals. However, caution should be exercised when proposing this diet based on current data. The data suggests that despite a regular exercise program, subjects on a LCHF diet showed a significant increase in TC and LDL-C during the 10-week intervention, possibly due to the higher intake of SFA and reduced intake in fiber. When combined with endurance exercise, the carbohydrate rich diets led to reduced levels of TC and LDL-C. However, the reduction in GI had a positive effect on the change in TG, while the high GI resulted in a decrease in HDL-C.

In summary, the data suggests that in active individuals, a diet low in carbohydrates and high in fat may lead to unfavorable alterations in blood lipid levels, while a diet rich in carbohydrates does not have such a detrimental effect on blood lipids. Additionally, reducing the glycemic index of consumed carbohydrates may result in a favorable change in TG concentration. Based on the findings of this study, it is recommended that active individuals who engage in regular exercise should be mindful of the potential impact of their diet on blood lipid levels. It would be beneficial for future research to consider the glycemic index when comparing the effects of a low- and high-carbohydrate diet on blood lipid levels, in order to gain further insights.

## Data Availability

The raw data supporting the conclusions of this article will be made available by the authors, without undue reservation.
